# Detection of Dimethyl Methyl Phosphonate by Silica Molecularly Imprinted Materials

**DOI:** 10.3390/nano13212871

**Published:** 2023-10-30

**Authors:** Xuming Wang, Xin Li, Qiang Wu, Yubin Yuan, Weihua Liu, Chuanyu Han, Xiaoli Wang

**Affiliations:** 1 Department of Microelectronics, Xi’an Jiaotong University, Xi’an 710049, China; xmwang_zw@126.com (X.W.); hanchuanyu@mail.xjtu.edu.cn (C.H.); 2 School of Physics, Xi’an Jiaotong University, Xi’an 710049, China

**Keywords:** gas sensor, molecularly imprinted materials, dimethyl methyl phosphonate, surface acoustic wave

## Abstract

In recent years, the increasing severity of chemical warfare agent threats to public safety has led to a growing demand for gas sensors capable of detecting these compounds. However, gas sensors used for the detection of chemical warfare agents must overcome limitations in sensitivity, selectivity, and reaction speed. This paper presents a sensitive material and a surface acoustic gas sensor for detecting dimethyl methyl phosphonate. The results demonstrate that the sensor exhibits good selectivity and could detect 80 ppb of dimethyl methyl phosphonate within 1 min. As an integral component of the sensor, the microstructure and adsorption mechanism of silica molecular imprinting material were studied in detail. The results show that the template molecule could significantly affect the pore volume, specific surface area, and hydroxyl density of mesoporous materials. These properties further affect the performance of the sensor. This study provides a valuable case study for the design of sensitive materials.

## 1. Introduction

Sarin is a volatile organic compound containing phosphorus and oxygen that can be used as a chemical weapon [1]. Sarin interacts with cholinesterase immediately after entering the body, rendering it incapable of hydrolyzing acetylcholine. This leads to the accumulation of acetylcholine, which in turn can lead to neurological problems and, in severe cases, death. Because sarin is easy to hide, it is often used as a terrorist weapon and poses a threat to public safety. Sarin sensors can effectively detect sarin and, therefore, become the most important means of early warning. Therefore, there is an urgent need to develop sarin sensors with fast response, high sensitivity, and good stability for early warning. It should be noted that usually in research, low-toxicity dimethyl methyl phosphonate (DMMP) is used as a simulant of sarin in consideration of the safety and health of researchers.

Sarin detection methods include chromatography [2], mass spectrometry [3], infrared spectrometry [4], ion mobility spectrometry [5,6], electrochemical sensors, metal oxide sensors [7], and surface acoustic wave (SAW) gas sensors. The SAW gas sensors show potential application prospects due to their advantages of high sensitivity, small size, and low cost. A SAW gas sensor consists of a piezoelectric substrate patterned with two interdigital transducers and a gas-sensitive film coated on the SAW propagation path. The absorption of target gas in the sensitive film will modulate SAW velocity and correspondingly change the phase or amplitude of the sensor output signal. According to this principle, the sensitivity and selectivity of the SAW gas sensor are mainly determined by the properties of the sensitive film. The sensitive materials available include hydrogen-bonded acidic compounds [8,9,10,11], polyepichlorohydrin, ethyl cellulose [12,13], metal oxide powders, and metal–organic framework (MOF) compounds [14]. These materials have excellent properties but are less designable than molecularly imprinted materials.

As a recent and rapidly evolving technology, molecular imprinting technology (MIT) has the benefits of a flexible approach and strong adsorption selectivity [15,16]. MIT uses DMMP molecules as templates to synthesize microporous materials with specific geometries. This porous material can efficiently and selectively adsorb DMMP molecules, making it an ideal sensitive material for SAW sensors. For sensitive materials used in SAW gas sensors, in order to improve sensitivity, low-density materials are usually preferred as sensitive films, such as carbon materials, alumina, and silica. Due to the advantages of silica in cost and preparation technology, it is suitable for preparing molecularly imprinted materials.

This study presents a mesoporous SiO_2_ molecularly imprinted polymer (MIP), which utilizes DMMP as the template for synthesis. The corresponding sensor has remarkable selectivity and sensitivity and achieves rapid detection of 80 ppb DMMP within 1 min. The sensitivity of the sensor is attributed to its high specific surface area and hydroxyl density. Moreover, the pore property and hydroxyl density of the sensitive material can be easily modulated by the content of the template DMMP. The large pore volume facilitates efficient diffusion of DMMP into the interior of the material, resulting in a fast response. These findings provide a compelling case for developing and designing materials with high sensitivity.

## 2. Materials and Methods

### 2.1. SAW Device Manufacturing

A SAW delay line structure with 200 nm thick aluminum interdigital transducers (IDTs) was fabricated on a 128° YX LiNbO_3_ wafer using a standard ultraviolet photolithography process. The delay line length between the IDTs was 2.56 mm. The designed SAW device operation frequency was 152 MHz, and the SAW velocity on the LiNbO_3_ wafer was 3890 m/s; thus, the corresponding wavelength λ was 25.6 μm. The apertures of the two IDTs were 100 λ, and their lengths were 130 λ and 40 λ, respectively. The prepared device was attached to a package base and coated with organic silicon rubber on the edge to reduce interference from reflected waves. Finally, the IDTs are connected to the pins of the base via wire bonding.

### 2.2. Synthesis of SiO_2_ MIPs

The reagents utilized in this work are listed below. Analytical reagent DMMP and ethanol were procured from Aladdin, while Macklin provided acetone, tetraethyl orthosilicate (TEOS), and hydrochloric acid (36%). Prior to preparation, a 0.1 mol/L dilute hydrochloric acid was produced by distilled water and hydrochloric acid. In order to study the effect of template DMMP on the performance of sensitive materials and gas sensors, 5 samples were arranged here for testing. Table 1 outlines the recipe for sensitive materials.

**Figure 1:**
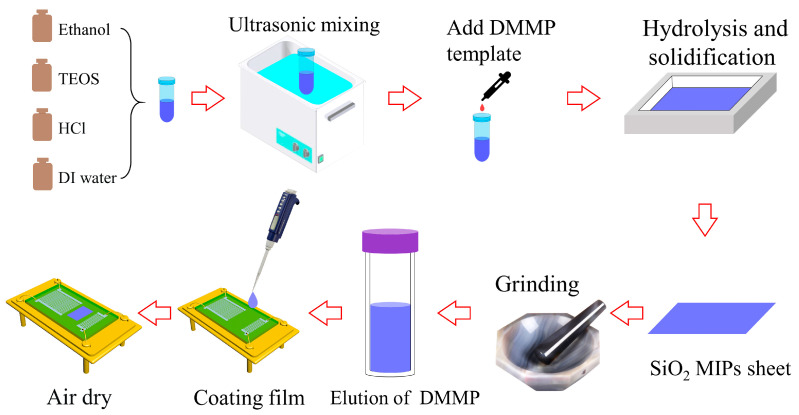
The preparation process of the SAW gas sensor.

The S4 sample exhibited exceptional performance; thus, it was chosen as a representative example to demonstrate the detailed preparation process. A polyethylene plastic tube was used to combine 1260 μL of TEOS, 655 μL of ethanol, 205 μL of distilled water, and 5.62 μL of diluted hydrochloric acid (0.1 mol/L). After an hour of ultrasonic blending, 75 μL of DMMP was added, and the mixture was swirled for 5 min. The mixture was poured onto a square Teflon dish and covered with aluminum foil to minimize DMMP evaporation. After approximately 72 h, SiO_2_ MIP sheets were obtained by this process. These sheets were then ground into approximately 1 µm powder by placing them in an agate mortar along with 1 mL of ethanol. During the grinding process, a small sample was collected to measure the size of the powder using a light microscope. Grinding ceased when particles close to 1 µm in size were observed in the powder sample. To remove any remaining DMMP from the SiO_2_ powder, it was transferred into a polypropylene centrifuge tube and soaked in 30 mL of ethanol for five minutes before being separated through centrifugation. This step was repeated three times to obtain an ethanol-dispersed SiO_2_ MIP suspension with a concentration of approximately 11.2 mg/mL. The gas sensor was fabricated by dropping 2 μL suspension onto the SAW device using a pipette gun, followed by subsequent drying. The overall preparation procedure is illustrated in Figure 1.

### 2.3. Material Characterization

The scanning electron microscope (SEM) was a Regulus 8100 (Hitachi, Hitachinaka, Japan), and the X-ray photoelectron spectrometer (XPS) was a Thermo ESCALAB 250xi (Thermo, Waltham, MA, USA). The atomic force microscope (AFM) image is obtained by Bruker Dimension Icon (Bruker, Billerica, MA, USA), and the X-ray diffraction (XRD) diagram was obtained by Bruker D8 advance (Bruker, Billerica, MA, USA). The Raman spectra were obtained by RENISHAW 2000 (RENISHAW, Singapore), and the infrared absorption spectra were obtained by Shimadzu UV-3600 UV-VIS-NIR (Shimadzu, Kyoto, Japan). The surface areas of SiO_2_ MIPs were measured by Micromeritics ASAP2020 (Micromeritics, Norcross, GA, USA), and the solid nuclear magnetic resonance (NMR) spectrum was obtained from BRUKER AVANCE III HD 400M (Bruker, Billerica, MA, USA). The thickness of the sensitive film was measured using the KLA Tencor D-100 profiler (KLA Corporation, Milpitas, CA, USA). The network analyzer is ADVANTEST R3765CG (Advantest, Tokyo, Japan).

The samples before and after the elution of DMMP were studied, and the suffixes D and ND were used to represent the two samples. For example, S1 D represents the S1 sample containing the template molecule DMMP inside the material, and S1 ND represents the S1 sample after the template DMMP has been removed.

### 2.4. Sensor Test

**Figure 2:**
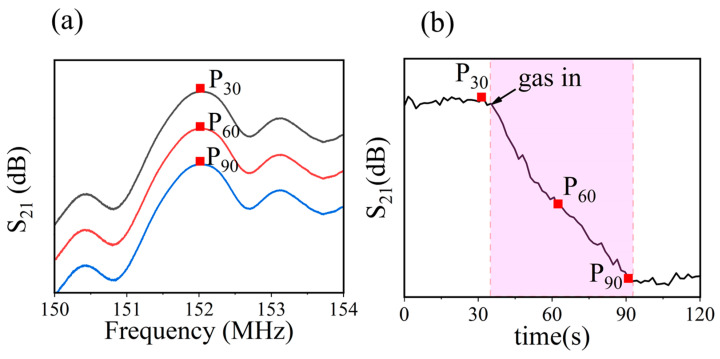
(**a**) Schematic diagram of extraction of characteristic signals from the S_21_ curve. (**b**) The response curve of the gas sensor.

A test system was used to evaluate the performance of the SAW gas sensor. The sensors were positioned in a 27 L chamber and connected to a computer-controlled network analyzer for measuring transmission properties. The liquid DMMP was injected into a small heater using a micro syringe, which is an evaporator to generate DMMP gas. Details of the test system are shown in Figure S1 of Supporting Information. After the adsorption of DMMP by sensitive materials, the increased mass of sensitive materials results in a change in the S_21_ curve of the SAW sensor. The maximum value P_t_ (t representing time) is extracted from the S_21_ curve as the output signal of the SAW sensor. As shown in Figure 2a, P_30_ represents the maximum value of the S_21_ curve of the SAW sensor at 30 s. These output signals constitute the dynamic response curve of the sensor, as shown in Figure 2b.

### 2.5. Material Calculation

Here, the adsorption mechanism of SiO_2_ MIPs was investigated using first-principles calculations. The program was ORCA 5.0.3, functional r2SCAN-3c for geometric optimization, ωB97M-V functional and def2-TZVP basis set with dispersion function for binding energy calculation [17], Multiwfn [18] for electrostatic potential (ESP) [19,20,21] and independent gradient model based on Hirshfeld partition (IGMH) [22]. The ESP of silanol and DMMP was calculated to identify potential active sites. The negative and positive potentials of ESP are shown in red and blue, respectively. Then, the strength and type of the intermolecular force are analyzed and shown by the IGMH. Strong attractive interaction (hydrogen bond) is shown in blue, weak attractive interaction (van der Waals force) is shown in green, and repulsive interaction is shown in red. According to the molar ratio between TEOS and DMMP of S4 in the recipe, a molecular cage containing 8 Si atoms is constructed to approximately replace the amorphous SiO_2_ MIPs, which is used to calculate the binding energy between the material and DMMP.

## 3. Results

### 3.1. Characterization Results

**Figure 3:**
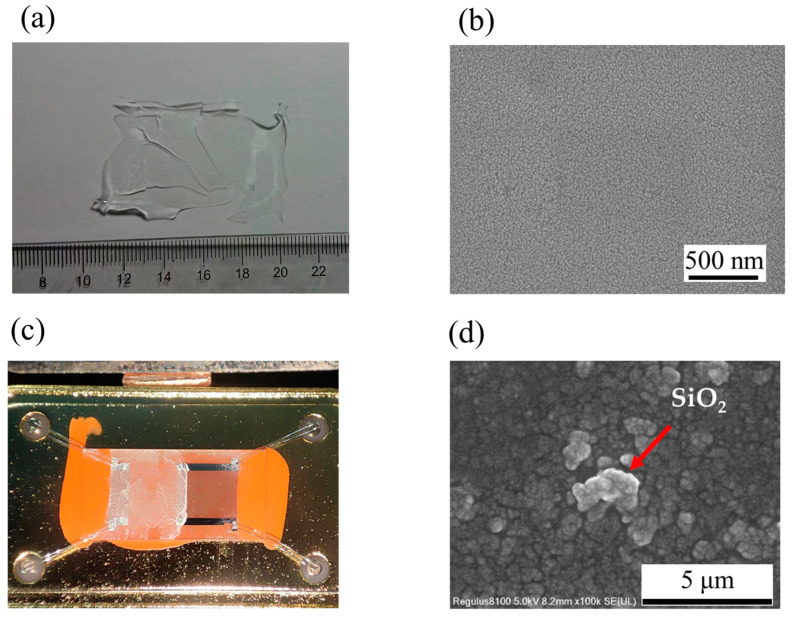
(**a**) A lamellar SiO_2_ MIP. (**b**) SEM image of lamellar SiO_2_ MIP. (**c**) The optical image of SiO_2_ MIP powder on a SAW sensor. (**d**) SEM image of SiO_2_ MIP powder.

The prepared lamellar SiO_2_ MIPs are shown in Figure 3a, while the SEM image of the SiO_2_ sheet is presented in Figure 3b. It can be confirmed that the prepared SiO_2_ MIPs are materials containing micropores, and the rough interface presented on the surface of the material can be observed by AFM, as shown in Figure S2. The optical image of SiO_2_ MIPs coated on the SAW gas sensor is shown in Figure 3c. The SEM images in Figure 3d indicate that the particle sizes of SiO_2_ MIPs range from 1 to 3 μm. The average thickness of the sensitive film was 0.8 μm with a standard deviation of 0.55 μm. Additional details are provided in Figure S3 in the Supporting Information.

**Figure 4:**
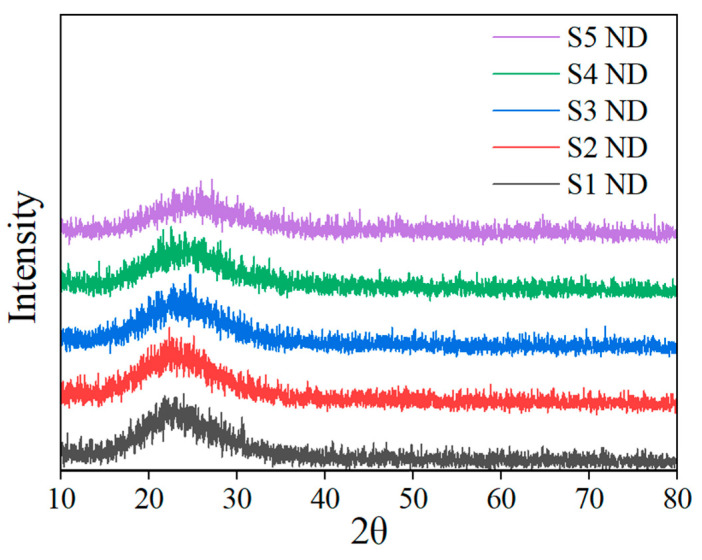
XRD of 5 SiO_2_ MIP samples.

XRD was employed to investigate the crystal structure of SiO_2_ powders prepared with five formulations. No significant diffraction peaks appear in the XRD results, indicating that the prepared SiO_2_ powder is amorphous [23], as shown in Figure 4. It should be noted that for material calculations, there is a significant difference between the models of amorphous SiO_2_ and crystalline SiO_2_. For crystalline SiO_2_, the supercell model can be used for calculation. For amorphous SiO_2_ materials, a specific model needs to be established. In the follow-up study, an amorphous model of SiO_2_ was established. 

**Figure 5:**
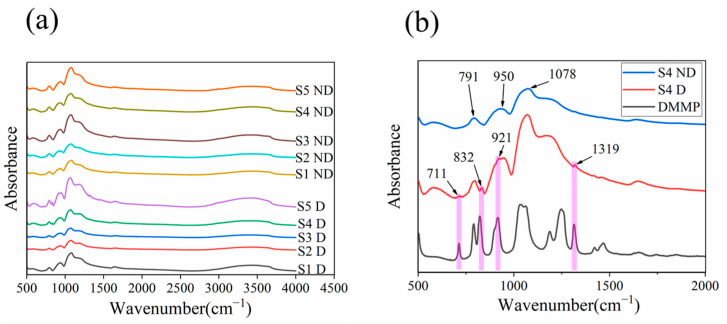
(**a**) Infrared absorption spectra of SiO_2_ samples. (**b**) Fine spectrum in S4 sample.

The infrared absorption spectra of SiO_2_ powders are presented in Figure 5a. Notably, distinct absorption peaks attributed to DMMP can be observed in S4 D and S5 D. In Figure 5b, the fine absorption spectra of S4 ND, S4 D, and liquid DMMP are depicted. The presence of characteristic peaks at 791 cm^−1^, 950 cm^−1,^ and 1078 cm^−1^ in S4 ND indicates the SiO_2_ material. Specifically, the peak at 950 cm^−1^ corresponds to the Si–OH functional group present in abundance within the prepared SiO_2_ material [24,25]. On the other hand, the peaks observed at 711 cm^−1^, 832 cm^−1^, 921 cm^−1,^ and 1319 cm^−1^ in S4 D correspond to specific characteristic peaks associated with DMMP. The successful immobilization of DMMP during the preparation of SiO_2_ MIPs is demonstrated by the infrared absorption spectra. In order to confirm the fixation of template molecules in SiO_2_, besides infrared absorption spectra, Raman spectra and the method of replacing template molecules were used to study this process. The results of these experiments are summarized in the Supporting Information. SiO_2_ MIPs were prepared using Rhodamine 6G (R6G) as a template, and the material was placed in water, and the red color gradually faded. This strongly proves that the SiO_2_ MIP is a porous material, and R6G could be diffused from the inside of the material. Figure S4 shows this process. Figure S5 shows the Raman spectrum results of SiO_2_ MIPs. Figure S6 shows the fine infrared spectra of 5 samples without elution of DMMP.

**Figure 6:**
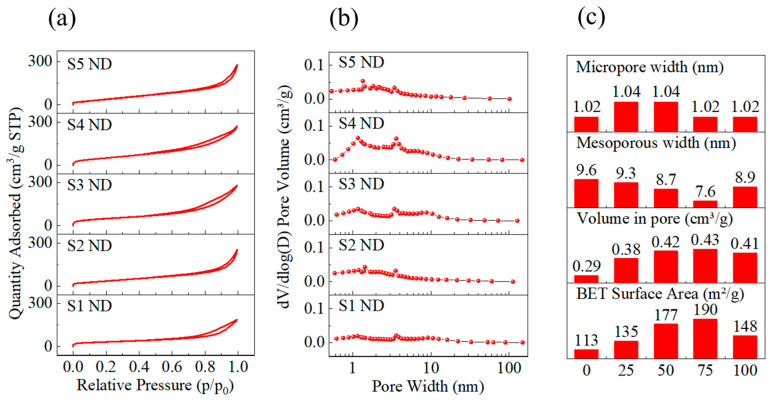
(**a**) Isothermal adsorption and desorption curve of SiO_2_ MIPs. (**b**) Pore width distribution of SiO_2_ MIPs. (**c**) Specific surface area, pore volume, and pore width of SiO_2_ MIPs.

To investigate the pore and surface hydroxyl properties of SiO_2_, the specific surface area, pore width, and surface hydroxyl groups of SiO_2_ were examined using BET analysis and solid-state NMR. The absorption-desorption isotherm of the SiO_2_ MIPs is presented in Figure 6a, while all 5 samples showed IVa-type isotherms, which confirmed the existence of a large number of mesoporous in the material. Figure 6b illustrates the distribution of pore width in SiO_2_ MIPs. With the increase in DMMP content in the recipe, the pore volume within 10 nm increased significantly. However, when 100 µL DMMP was added, the pore volume decreased. The pore properties are summarized in Figure 6c. The specific surface area of the SiO_2_ MIPs increased with increasing DMMP in the recipe, from 113 m^2^/g (S1 ND, DMMP 0 µL) to 190 m^2^/g (S4 ND, DMMP 75 µL) and then decreased to 148 m^2^/g (S5 ND, DMMP 100 µL). The pore volume of SiO_2_ MIPs increases from 0.29 cm^3^/g (S1) to 0.43 cm^3^/g (S4 ND) and then drops to 0.41 cm^3^/g (S5 ND) with an increase in DMMP. The average mesoporous width of the material changed from 9.6 nm (S1 ND) to 7.6 nm (S4 ND) and subsequently increased to 8.9 nm (S5 ND), showing that the quantity of mesoporous pores grew as DMMP increased. The five samples had micropore widths of 1.02 nm to 1.04 nm. The addition of DMMP had no effect on the micropore width of the sample but significantly increased the mesopore volume and specific surface area of the material.

**Figure 7:**
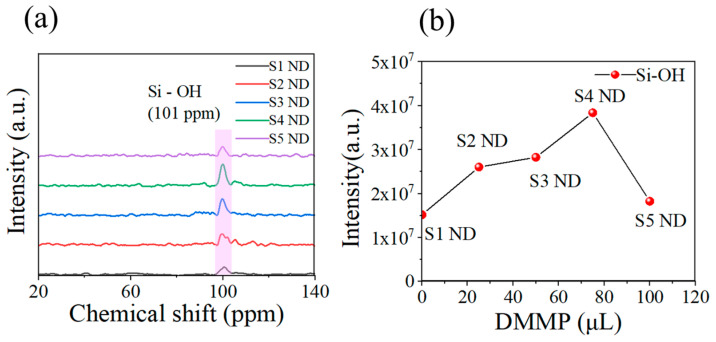
(**a**) NMR spectra of SiO_2_ MIPs. (**b**) Relationship between Si-OH peak intensity and DMMP addition.

The solid-state ^29^Si NMR spectra of five samples are depicted in Figure 7a. In the NMR spectrum, a peak appears at 101 ppm, which confirms the presence of the Si–OH structure [26]. Each sample used for testing solid-state NMR has a mass of 40 mg. The higher the number of hydroxyl groups in the sample, the higher the corresponding peak intensity. The relationship between peak intensity and DMMP content in the recipe is shown in Figure 7b. With the increase in DMMP, the number of hydroxyl groups in SiO_2_ first increased and then decreased, reaching a maximum value of 75 µL.

**Figure 8:**
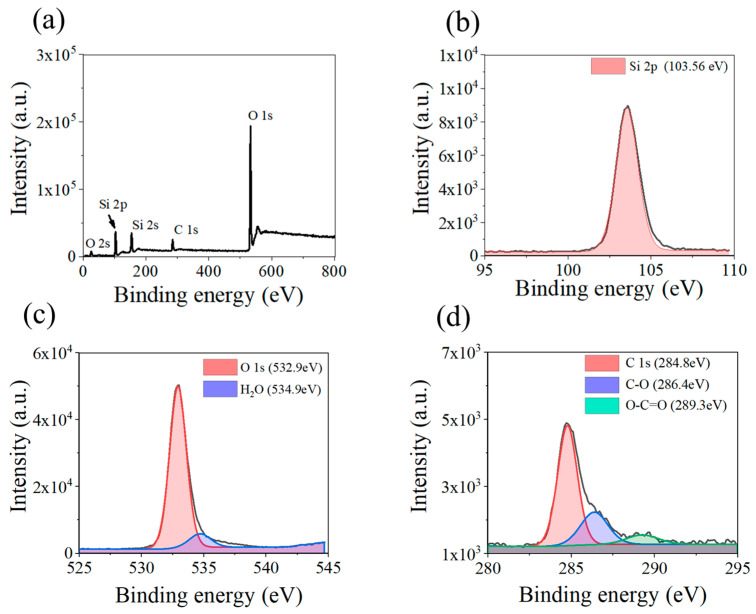
(**a**) XPS spectra of SiO_2_ MIPs. (**b**) High-resolution Si spectrum. (**c**) High-resolution O spectrum. (**d**) High-resolution C spectrum.

The elemental composition of the S4 ND samples was examined using XPS, as shown in Figure 8. These findings indicate that the major components of the SiO_2_ samples are silicon, oxygen, and trace amounts of carbon. The high-resolution O spectrum showed that SiO_2_ adsorbed a certain amount of water. In Figure 8d, XPS shows that carbon exists mainly as C–O and O–C=O [27]. In both functional groups, the O atom is negatively charged and easily interacts with the hydroxyl group in SiO_2_. It can be considered that the carbon is derived from the organic residue in the preparation process. 

**Figure 9:**
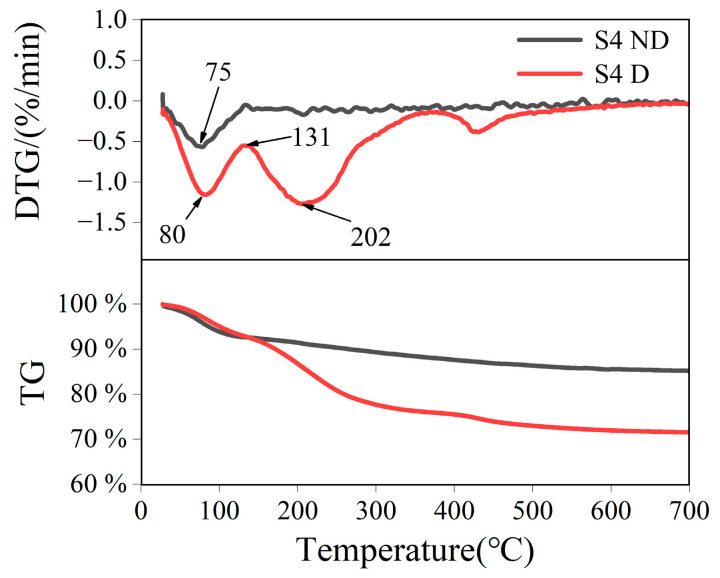
TG analysis curves of SiO_2_ MIPs.

The thermogravimetric (TG) curves of SiO_2_ MIPs are presented in Figure 9. For the S4 D sample, the decreasing mass from room temperature to 131 °C can be attributed to the evaporation of adsorbed water. With the further increase in temperature, obvious mass loss occurs at 202 °C. This is attributed to the volatilization of DMMP inside the material. The mass loss at 400 °C may be related to the high boiling compound adsorbed by the material. In contrast, no significant mass loss due to DMMP volatilization was observed in S4 ND samples. When the temperature is raised from room temperature to 131 °C, the mass loss of the S4 ND sample is 8%, which can be attributed to the evaporation of adsorbed water inside the material. As the temperature increases, the hydroxyl interaction on SiO_2_ is converted into water, resulting in a gradual decline in the total mass from 92% to 85%.

### 3.2. Performance of Gas Sensor 

**Figure 10:**
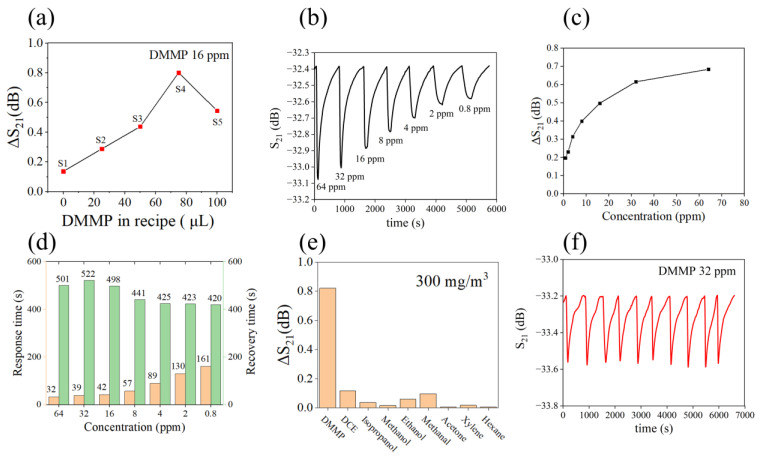
(**a**) Responsiveness of five sensors to DMMP at 16 ppm. (**b**) Dynamic response curves of the S4 sensor. (**c**) Relationship between gas concentration and response. (**d**) Sensor response time and recovery time. (**e**) The selectivity of the sensor. (**f**) The repeatability of the sensor.

**Figure 11:**
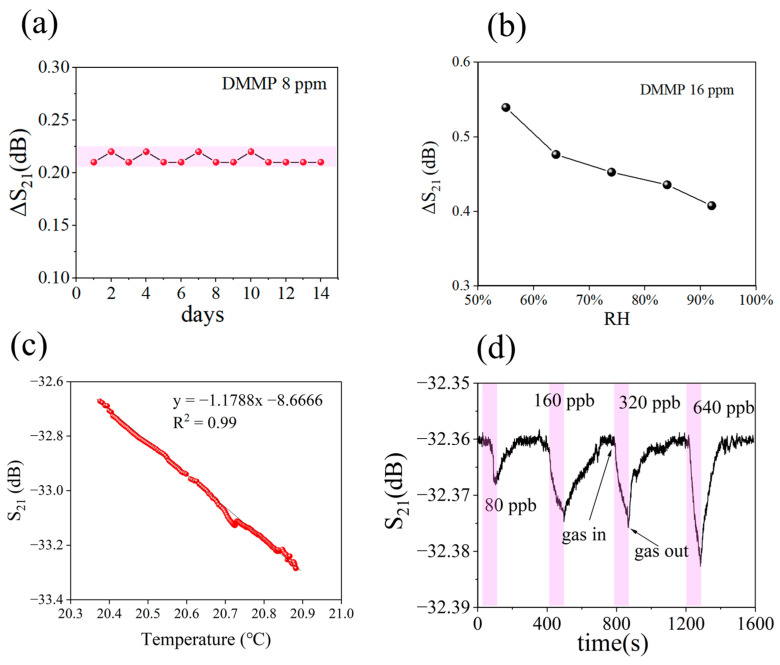
(**a**) Aging properties of sensors. (**b**) Response of sensor at different relative humidity. (**c**) The effect of temperature on the baseline of the sensor. (**d**) Sensor response to ultra-low concentrations of DMMP.

The sensor was tested following exposure to DMMP vapor, and the test time was 3 min. The response, ΔS_21_, was calculated as the difference between S_21_ at 180 s and S_21_ at the beginning of the test. Initially, sensor response tests were performed on five samples, and it was determined that the S4 formulation exhibited the highest sensitivity. Figure 10a presents the test results, while detailed information can be found in Figure S7 in the Supporting Information. The subsequent sensors tested were all coated with S4 ND as a sensitive material. Figure 10b illustrates the sensor response under various concentrations of DMMP. The correlation between DMMP concentration and sensor response is depicted in Figure 10c. Additionally, Figure 10d presents the response time and recovery time of the sensor for different concentrations of DMMP. These findings indicate a reduction in response time with increasing DMMP. Specifically, a concentration of 0.8 ppm DMMP resulted in a response time of 161 s. Furthermore, Figure 10e demonstrates selectivity testing on nine gases at a concentration of 300 mg/m^3^, while detailed information can be found in Figure S8. SiO_2_ MIPs exhibited selective adsorption towards DMMP. Figure 10f shows repeated testing of the sensor over 10 cycles at a concentration of 32 ppm DMMP. The aging performance of the sensor at 8 ppm DMMP is shown in Figure 11a. The SiO_2_ MIPs demonstrate excellent robust performance over 14 days. Figure 11b shows the sensor response to 16 ppm DMMP at various relative humidity (RH). The responsiveness of the sensor decreases with increasing humidity. The response curves of the sensor at different humidity are shown in Figure S9. Figure 11c shows how temperature affects the sensor baseline in clean air. The results show that the sensor baseline has a linear relationship with temperature, and the fitted curve R^2^ value is 0.99. This indicates that temperature compensation can be used to solve sensor baseline drift. Figure 11d shows the response curve of the sensor to ultra-low concentrations of DMMP. The test time was 1 min, and the sensor was able to detect a minimum concentration of 80 ppb DMMP.

A comparison of our study with previous studies is shown in Table 2. It can be seen that our sensor performs well in terms of response speed and detection limit.

## 4. Discussion

**Figure 12:**
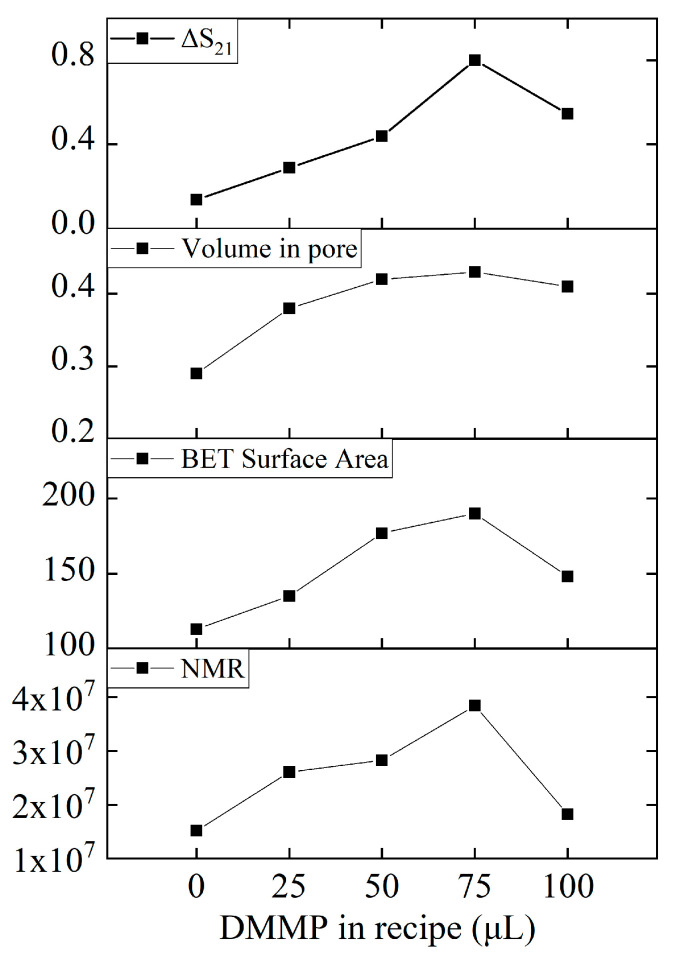
Relationship of the amount of DMMP added to the responsiveness, pore volume, specific surface area, and NMR intensity.

Sensor responsiveness, pore volume, specific surface area, and intensity of 101 ppm peak acquired by NMR were summarized in relation to DMMP content in the recipe, revealing a consistent pattern as shown in Figure 12. With the increase in DMMP content, the responsiveness of the sensor, the specific surface area and pore volume of the material, and the surface hydroxyl density of the material all increased first and then decreased. This shows that DMMP as a template can modulate the formation of mesoporous and the number of hydroxyl groups in the material.

**Figure 13:**
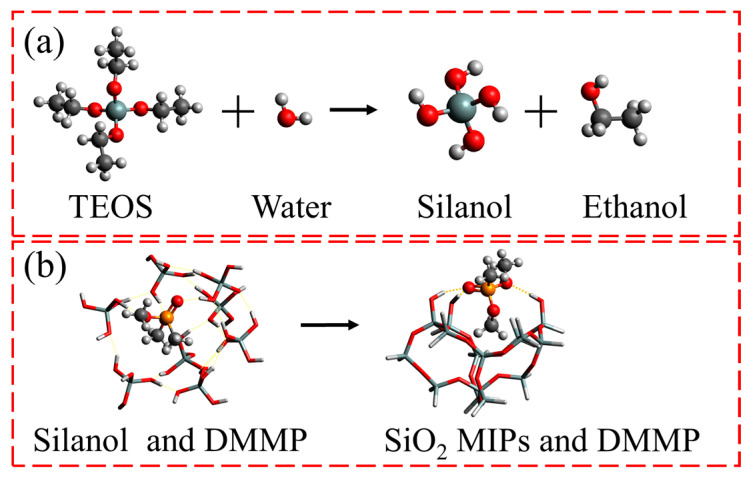
(**a**) Hydrolysis of TEOS. (**b**) The polycondensation reaction process of silane alcohols.

Under the catalysis of hydrochloric acid, TEOS and water are hydrolyzed to produce silanol and ethanol [34,35]. These chemical processes are shown in Figure 13a. In the silanol–DMMP mixture, some silanols can form hydrogen bonds with DMMP, which is attributed to the electrostatic interaction between the hydroxyl group in the silanol and the oxygen in DMMP. The hydroxyl groups between silanols then undergo a gradual polycondensation process to generate SiO_2_. However, since a portion of the hydroxyl group has formed a hydrogen bond with the oxygen of the DMMP, this portion of the hydroxyl group is maintained and does not participate in the polycondensation. These residual hydroxyl groups form active sites in SiO_2_ MIPs and aid in the identification and trapping of DMMP. The volume of pores and the number of hydroxyl groups in SiO_2_ increases with DMMP. However, too much DMMP causes too many hydroxyl groups to form hydrogen bonds, reducing the number of hydroxyl groups involved in polycondensation reactions and, as a result, inhibiting the synthesis of SiO_2_. These chemical processes are shown in Figure 13b. To identify the two molecules, DMMP is represented by a ball-and-stick model, whereas silanol is represented by a stick model.

**Figure 14:**
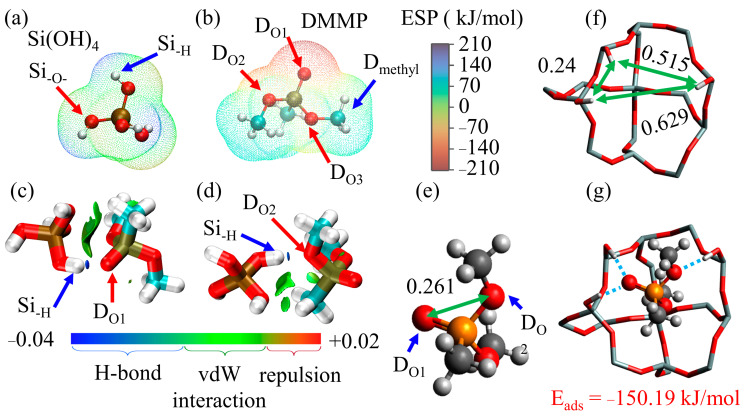
(**a**) ESP of Si (OH)_4_. (**b**) ESP of DMMP. (**c**) Hydrogen bond between Si_–H_ and D_O1_. (**d**) Hydrogen bond between Si_–H_ and D_O2_. (**e**) The distance between D_O1_ and D_O2_ of DMMP is 0.261 nm. (**f**) The distance of three Si_–H_ in SiO_2_ MIPs. (**g**) One DMMP forms hydrogen bonds with three hydroxyl groups.

The interaction between DMMP and silanol was further confirmed by first-principles calculation. Si_–H_ represents the hydrogen of the hydroxyl group in silanol, and Si_–O–_ represents its oxygen. In DMMP, D_Ox_ stands for the O atom at position x, and D_methyl_ is the methyl group. The ESP of silanol is depicted in Figure 14a, where Si_–H_ exhibits a positive potential, and Si_–O–_ exhibits a negative potential. The ESP of DMMP is shown in Figure 14b, with D_methyl_ exhibiting a positive potential and D_O1_ and D_O2_ exhibiting negative potentials. It can be deduced that Si_–H_ tends to attract D_Ox_, while Si_–O–_ tends to attract D_methyl_. The IGMH of DMMP and silanol are depicted in Figure 14c,d. In Figure 14c, the hydroxyl group forms a hydrogen bond with the oxygen atom of DMMP (shown in blue), while the remaining interactions are attributed to van der Waals forces (shown in green). The binding energies of the Si_–H_ bonds with D_O1_ and D_O2_ are −52.23 kJ/mol and −34.96 kJ/mol, respectively. It is difficult to form strong bonds between D_O3_ and Si_–H_, as is the case between Si_–O–_ and D_methyl_. Detailed calculation results are in Table S2 of Supporting Information.

Several physical effects, including mass loading effects, viscoelastic effects, and acoustoelectric effects, are believed to be responsible for the variation of the sensitivity parameters of the SAW gas sensor. Since SiO_2_ MIPs are granular insulating materials, mass loading effects are the main mechanism. That is, with the adsorption of gas molecules, the mass of the sensitive film increases, changing the S_21_ of the SAW sensor. The adsorption mechanism was verified by calculation.

Calculations have demonstrated that DMMP can form stable structures with up to three silanol groups and retain them after polymerization; that is, two hydroxyl groups formed a stable hydrogen bond with D_O1_, whereas one hydroxyl group formed a stable hydrogen bond with D_O2_, as illustrated in Figure 14g. These three hydroxyl groups in the micropores of SiO_2_ record the geometry of the DMMP. The distance between D_O1_ and D_O2_ in DMMP is 0.261 nm, as shown in Figure 14e. The spacing between the three Si_–H_ is 0.24 nm, 0.515 nm, and 0.629 nm, as shown in Figure 14f, which allows SiO_2_ MIPs to identify and capture DMMP. Other molecules are difficult to capture by SiO_2_ MIPs due to the geometric mismatch. The three hydroxyl groups form hydrogen bonds with D_O1_ and D_O2_ of the DMMP, with a total binding energy E_ads_ of −150.19 kJ/mol. Detailed calculation results are shown in Table S3 of Supporting Information. This binding energy is roughly equal to the sum of the binding energies of three Si_–H_ with D_O1_ and D_O2_. This result supports the adsorption mechanism of SiO_2_ MIPs to a certain extent.

## 5. Conclusions

In conclusion, a highly sensitive SAW gas sensor and a sensitive film preparation method for DMMP detection are developed in this work. Amorphous mesoporous SiO_2_ MIP materials were prepared by TEOS hydrolysis polymerization using DMMP as a template. The content of the DMMP template significantly affects the specific surface area, pore volume, and the number of hydroxyl groups on the surface of the material. With SiO_2_ MIPs as the sensitive material of the SAW gas sensor, the detection of 80 ppb DMMP was realized. The first principles calculation revealed the interaction and binding energy between the SiO_2_ MIPs and DMMP and confirmed two important active sites in the DMMP molecule, which are the two O atoms of DMMP. Through systematic experiments and theoretical analysis, the developed SAW gas sensor has a broad application prospect in the detection of sarin.

## Figures and Tables

**Table 1:** The recipe for preparing samples.

Recipe	S1	S2	S3	S4	S5
DMMP (μL)	0	25	50	75	100
Ethanol (μL)	655				
TEOS (μL)	1260				
Water (μL)	205				
HCl (μL)	5.62				

**Table 2:** Comparison of this work with the previous works.

Frequency	Sensitive Material	Limit of Detection (DMMP)	Response Time	Ref.
300 MHz	molecularly imprinted polymer	0.1 ppm	300 s	[28]
434 MHz	polymethyl[3-(2-hydroxy) phenyl] siloxane	5 ppm	30 s	[29]
163 MHz	carbowax, polyethyleneimine, polyepichlorohydrin. etc.	0.04 ppm	1800 s	[30]
392 MHz	polyethyleneimine, polyepichlorhydrine, polyisobutylene	2.5 ppm	60 s	[31]
150 MHz	fluoroalcoholpolysiloxane (SXFA)	0.025 ppm		[32]
152 MHz	hexafluoroisopropanol (HFIP) modified carbon nanotubes	0.1 ppm	3 s	[33]
152 MHz	molecularly imprinted polymer (SiO_2_ MIPs)	0.08 ppm	60 s	this work

## Data Availability

The data supporting the findings of this study are available from the corresponding author upon reasonable request.

## Supplementary Materials

The following supporting information can be downloaded at https://www.mdpi.com/article/10.3390/nano13212871/s1, Figure S1. (a) The overall appearance of the test system. (b) The SAW sensor and fixture. (c) A fan and a heater on top of chamber. Figure S2. (a) The lamellar SiO_2_ MIPs material. (b) Morphology and defects of lamellar SiO_2_ surface. (c) High resolution image of lamellar SiO_2_. (d) AFM image of SiO_2_. Figure S3. (a) SiO_2_ MIPs dispersed by ethanol. (b) A sensor with SiO_2_ MIPs. (c) and (d) SEM image of SiO_2_ powder. (e,f) The thickness of the sensitive film along the red line. Figure S4. (a) SiO_2_ MIPs prepared using R6G as template. (b) The R6G in SiO_2_ MIPs diffuses from the material to the water. (c,d) The red color of SiO_2_ MIPs faded gradually. Figure S5. Raman spectra of SiO_2_ materials. Figure S6. Infrared absorption spectra of SiO_2_ MIPs. Figure S7. (a) Response curves of the sensors corresponding to the five formulations. (b) The relationship between concentration and response. Figure S8. The dynamic response curve of the sensor to different gases. Figure S9. Response curve of the sensor to DMMP at different humidity. Table S1. Mass concentration (300 mg/m^3^) corresponding volume concentration (ppm). Table S2. Binding energy between silanol and DMMP at different sites. Table S3. Binding energy of SiO_2_ and DMMP. Refs. [36,37,38] are cited in the Supplementary Materials.

## References

[1] Colović M.B., Krstić D.Z., Lazarević-Pašti T.D., Bondžić A.M., Vasić V.M. (2013). Acetylcholinesterase inhibitors: Phar-macology and toxicology. Curr. Neuropharmacol..

[2] Li Y., Du X.S., Wang Y., Tai H.L., Qiu D., Lin Q.H., Jiang Y.D. (2014). MEMS-based gas chromatography column for the analysis of chemical warfare agent(CWA) simulates. Appl. Mech. Mater..

[3] Terzic O., Swahn I., Cretu G., Palit M., Mallard G. (2012). Gas chromatography-full scan mass spectrometry determina-tion of traces of chemical warfare agents and their impurities in air samples by inlet based thermal desorption of sorbent tubes. J. Chromatogr. A.

[4] Phillips C.M., Tan H.W. (2010). FTIR Gas Analysis with Improved Sensitivity and Selectivity for CWA and TIC Detection. Chemical, Biological, Radiological, Nuclear, and Explosives (CBRNE) Sensing XI.

[5] Armenta S., Alcala M., Blanco M. (2011). A review of recent, unconventional applications of ion mobility spectrometry (IMS). Anal. Chim. Acta.

[6] Puton J., Namiesnik J. (2016). Ion mobility spectrometry: Current status and application for chemical warfare agents detection. Trac-Trends Anal. Chem..

[7] Patil L.A., Deo V.V., Shinde M.D., Bari A.R., Patil D.M., Kaushik M.P. (2014). Improved 2-CEES sensing performance of spray pyrolized Ru-CdSnO3 nanostructured thin films. Sens. Actuators B Chem..

[8] Kong L.T., Wang J., Fu X.C., Zhong Y., Meng F.L., Luo T., Liu J.H. (2010). p-Hexafluoroisopropanol phenyl covalently functionalized single-walled carbon nanotubes for detection of nerve agents. Carbon.

[9] Long Y., Wang Y., Du X.S., Cheng L.H., Wu P.L., Jiang Y.D. (2015). The Different Sensitive Behaviors of a Hydrogen-Bond Acidic Polymer-Coated SAW Sensor for Chemical Warfare Agents and Their Simulants. Sensors.

[10] Grate J.W. (2008). Hydrogen-Bond Acidic Polymers for Chemical Vapor Sensing. Chem. Rev..

[11] Grate J.W. (2000). Acoustic wave microsensor arrays for vapor sensing. Chem. Rev..

[12] Ballantine D.S., Rose S.L., Grate J.W., Wohltjen H. (1986). Correlation of surface acoustic wave device coating responses with solubility properties and chemical structure using pattern recognition. Anal. Chem..

[13] Grate J.W., Rose-Pehrsson S.L., Venezky D.L., Klusty M., Wohltjen H. (1993). Smart sensor system for trace organophos-phorus and organosulfur vapor detection employing a temperature-controlled array of surface acoustic wave sen-sors, automated sample preconcentration, and pattern recognition. Anal. Chem..

[14] Devkota J., Greve D.W., Hong T., Kim K.J., Ohodnicki P.R. (2020). An 860 MHz Wireless Surface Acoustic Wave Sensor with a Metal-Organic Framework Sensing Layer for CO_2_ and CH4. IEEE Sens. J..

[15] BelBruno J.J. (2019). Molecularly Imprinted Polymers. Chem. Rev..

[16] Holthoff E., Li L., Hiller T., Turner K. (2015). A molecularly imprinted polymer (MIP)-coated microbeam MEMS sensor for chemical detection. Chemical, Biological, Radiological, Nuclear, and Explosives (CBRNE) Sensing XVI.

[17] Goerigk L., Hansen A., Bauer C., Ehrlich S., Najibi A., Grimme S. (2017). A look at the density functional theory zoo with the advanced GMTKN55 database for general main group thermochemistry, kinetics and noncovalent interactions. Phys. Chem. Chem. Phys..

[18] Lu T., Chen F.W. (2012). Multiwfn: A multifunctional wavefunction analyzer. J. Comput. Chem..

[19] Lu T., Manzetti S. (2014). Wavefunction and reactivity study of benzo[a]pyrene diol epoxide and its enantiomeric forms. Struct. Chem..

[20] Manzetti S., Lu T. (2013). The geometry and electronic structure of Aristolochic acid: Possible implications for a frozen resonance. J. Phys. Org. Chem..

[21] Zhang J., Lu T. (2021). Efficient evaluation of electrostatic potential with computerized optimized code. Phys. Chem. Chem. Phys..

[22] Lu T., Chen Q.X. (2022). Independent gradient model based on Hirshfeld partition: A new method for visual study of in-teractions in chemical systems. J. Comput. Chem..

[23] Sun J.F., Xu Z.Q., Li W.F., Shen X.D. (2017). Effect of Nano-SiO_2_ on the Early Hydration of Alite-Sulphoaluminate Cement. Nanomaterials.

[24] Saravanan S., Dubey R.S. (2020). Synthesis of SiO_2_ Nanoparticles by Sol-Gel Method and Their Optical and Structural Properties. Rom. J. Inf. Sci. Technol..

[25] Ramalla I., Gupta R., Bansal K. (2015). Effect on superhydrophobic surfaces on electrical porcelain insulator, improved technique at polluted areas for longer life and reliability. Int. J. Eng. Technol..

[26] Lin C., Rüssel C., van Wüllen L. (2019). Phase Separation and Nanocrystallization in KF–ZnF_2_–SiO_2_ Glasses: Lessons from Solid-State NMR. J. Phys. Chem. B.

[27] Biesinger M.C. (2022). Accessing the robustness of adventitious carbon for charge referencing (correction) purposes in XPS analysis: Insights from a multi-user facility data review. Appl. Surf. Sci..

[28] Wen W., Shitang H., Shunzhou L., Minghua L., Yong P. (2007). Enhanced sensitivity of SAW gas sensor coated molecu-larly imprinted polymer incorporating high frequency stability oscillator. Sens. Actuators B Chem..

[29] Du X., Ying Z., Jiang Y., Liu Z., Yang T., Xie G. (2008). Synthesis and evaluation of a new polysiloxane as SAW sensor coatings for DMMP detection. Sens. Actuators B Chem..

[30] Matatagui D., Fernández M.J., Fontecha J., Santos J.P., Gràcia I., Cané C., Horrillo M.C. (2012). Love-wave sensor array to detect, discriminate and classify chemical warfare agent simulants. Sens. Actuators B Chem..

[31] Di Pietrantonio F., Benetti M., Cannatà D., Verona E., Palla-Papavlu A., Dinca V., Dinescu M., Mattle T., Lippert T. (2012). Volatile toxic compound detection by surface acoustic wave sensor array coated with chemoselective polymers deposited by laser induced forward transfer: Application to sarin. Sens. Actuators B Chem..

[32] Pan Y., Zhang G.W., Guo T.X., Liu X.L., Zhang C.H., Yang J.C., Cao B.Q., Zhang C., Wang W. (2020). Environmental characteristics of surface acoustic wave devices for sensing organophosphorus vapor. Sens. Actuators B Chem..

[33] Wu Q., Li X., Wang X.M., Yuan Y.B., Bu X.R., Wu H.Y., Li X., Han C.Y., Wang X.L., Liu W.H. (2022). High-performance p-hexafluoroisopropanol phenyl functionalized multi-walled carbon nanotube film on surface acoustic wave device for organophosphorus vapor detection. Nanotechnology.

[34] Zhuravlev L.T. (2000). The surface chemistry of amorphous silica. Zhuravlev model. Colloids Surf. A.

[35] Plotnichenko V.G., Sokolov V.O., Dianov E.M. (2000). Hydroxyl groups in high-purity silica glass. Inorg. Mater..

[36] Lafuente M., Sanz D., Urbiztondo M., Santamaria J., Pina M.P., Mallada R. (2020). Gas phase detection of chemical warfare agents CWAs with portable Raman. J. Hazard. Mater..

[37] Wang J., Duan G., Liu G., Li Y., Chen Z., Xu L., Cai W. (2016). Detection of dimethyl methylphosphonate by thin water film confined surface-enhanced Raman scattering method. J. Hazard. Mater..

[38] Lin Z., Wen B., Liu W., Fu W., Kong J., Li J. (2022). Rapidly Detection of Chemical Warfare Agent Simulants by Surface Enhanced Raman Spectroscopy. Spectrosc. Spectr. Anal..

