# Supplementary material for: Detection of Dimethyl Methyl Phosphonate by Silica Molecularly Imprinted Materials
Source: Nanomaterials (Basel). 2023 Oct 30;13(21):2871. doi: 10.3390/nano13212871 (PMC10648664; doi:10.3390/nano13212871)
Supplement: Supplementary file 1 [file nanomaterials-13-02871-s001.zip › nanomaterials-2652743-supplementary.pdf]

Supporting Information

Detection of Dimethyl Methyl Phosphonate by Silica Molecularly Imprinted Materials

Xuming Wang [1], Xin Li [1,*], Qiang Wu [1], Yubin Yuan [1], Weihua Liu [1], Chuanyu Han [1]

and Xiaoli Wang [2]

[1] Department of Microelectronics, Xi'an Jiaotong University, Xi'an 710049, China

[2] School of Physics, Xi'an Jiaotong University, Xi'an 710049, China

[*] Correspondence: lx@mail.xjtu.edu.cn

The performance of the SAW sensor was tested via a lab-made test system. Figure S1 (a) shows the test system. From top to bottom are acrylic chamber, multimeter (Keithley2000) and network analyzer. Figure S1 (b) shows the SAW sensor and a fixture. A thermal resistor is attached to the fixture as a temperature sensor, which is connected to the multimeter. The network analyzer and the multimeter are connected to the computer to obtain the response curve and temperature of the sensor. Figure S1 (c) shows the fan and evaporator.

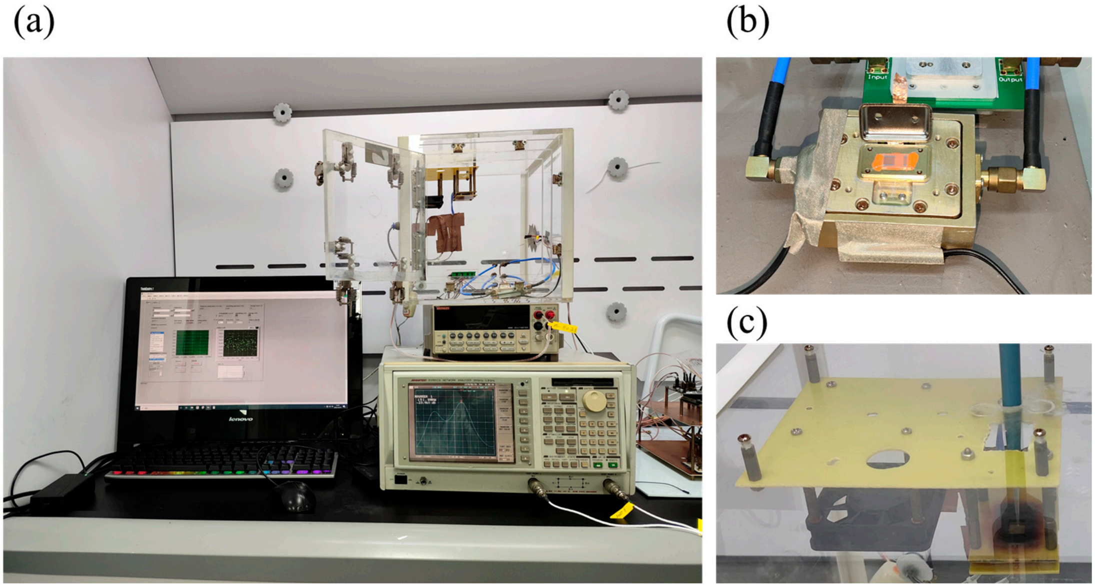

Figure S1. (a) The overall appearance of the test system. (b) The SAW sensor and fixture. (c) A fan and a heater on top of chamber.

The chamber is made of acrylic, and the length, width and height inside are 30 cm, so the volume is 27 L. A small heater with an operating temperature of 120℃ and a fan are installed at the

top of the chamber, and a fixture for the SAW sensor is installed at the bottom of the chamber. At the top of the chamber there is a hole with a lid facing the heater. Install the SAW sensor on the fixture and close the chamber door. Take a certain volume of liquid DMMP with a micro syringe, drop DMMP on the heater, and the liquid DMMP evaporates into a gas. Then turn on the fan to stir the DMMP gas evenly. In general, it takes about 3 s to get a uniform gas from the dripping of DMMP. The concentration of the DMMP vapor was controlled by the volume of injected liquid DMMP and evaluated using the following equation (S1).

$$c(\text{ppm}) = (V_{\text{DMMP}} \times \rho_{DMMP} \div M_{\text{DMMP}}) \times 0.907 \times 10^6 \tag{S1}$$

Where V_{DMMP}, ρ_{DMMP} and M_{DMMP} were the volume, density and molar mass of DMMP, respectively. The test system is housed in a fume hood. After the test process is complete, the chamber was opened and purged with air until the sensor returned to its initial state.

The lamellar SiO_2 MIPs were characterized using scanning electron microscope (SEM) and atomic force microscope (AFM), which revealing a visibly rough interface with a surface roughness Ra of 1.6 nm. Figure S2 shows this information. However, due to the insulating nature of SiO_2, the resolution of SEM is limited by its electrical conductivity, posing challenges in observing nanoscale pores on the surface of material. Consequently, the nitrogen adsorption desorption isotherm method was employed to investigate mesoporous.

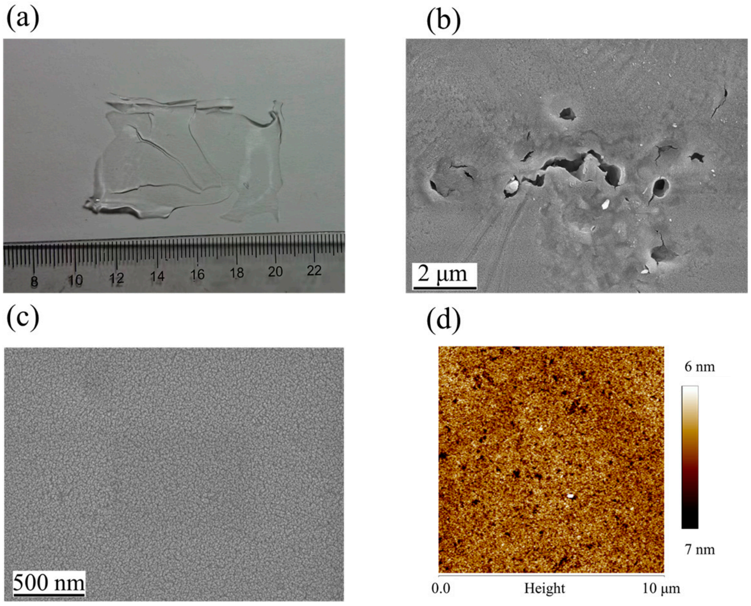

Figure S2.(a) The lamellar SiO_2 MIPs material.(b) Morphology and defects of lamellar SiO_2 surface.(c) High resolution image of lamellar SiO_2.(d) AFM image of SiO_2.

The lamellar SiO_2 was ground into powder and dispersed in ethanol to prepare a suspension, as shown in figure S3 (a). The sensor was prepared by taking a certain volume of suspension with a micro-syringe and applying drops on the SAW device, as shown in figure S3 (b). The particle size distribution of SiO_2 powder ranges from 1 to 3 μm, as shown in figure S3 (c) and (d). The thickness of the film was measured with a profiler, and the results were shown in figure S3 (e). The average thickness of the sensitive film was 0.8 μm with a standard deviation of 0.55 μm.

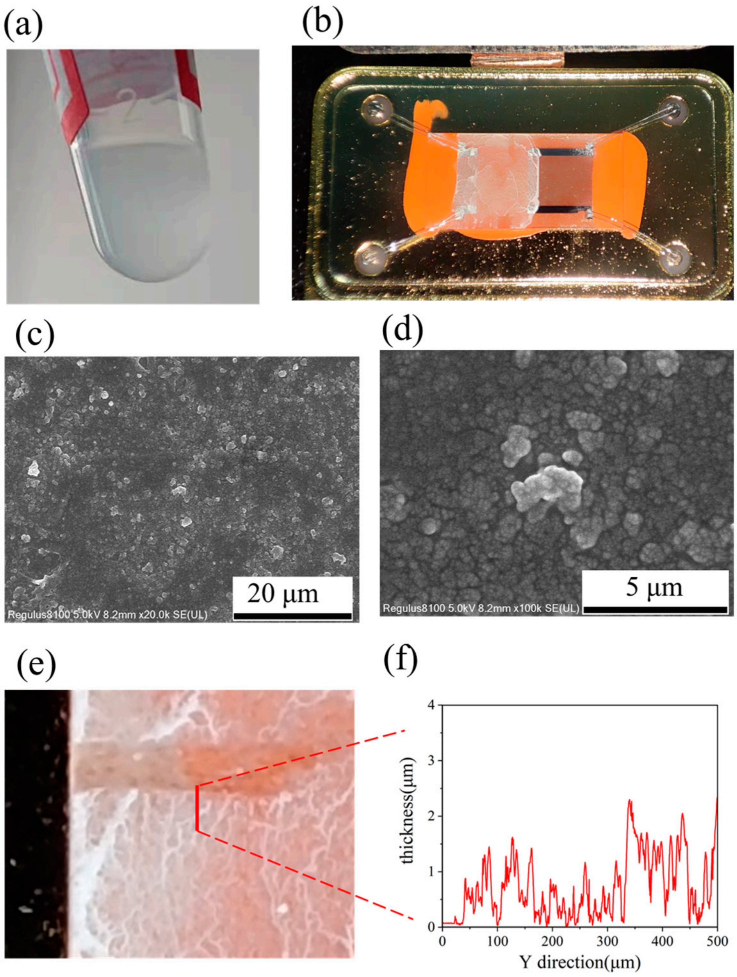

Figure S3. (a) SiO_2 MIPs dispersed by ethanol. (b) A sensor with SiO_2 MIPs. (c) and (d) SEM image of SiO_2 powder. (e) and (f) The thickness of the sensitive film along the red line.

The fixation of template molecules in materials was observed by various methods. The first method visually demonstrates the fixation and desorption process of template molecules in SiO_2 MIPs. SiO_2 MIPs was prepared using Rhodamine 6G (R6G) as the template molecule. The corresponding SiO_2 MIPs were prepared by adding 1260 μL TEOS, 655 μL ethanol, 205 μL distilled water, 5.62 μL diluted hydrochloric acid (0.1 mol/L) and 8mg R6G into the polyethylene plastic pipe. Since R6G appears red, the synthesized SiO_2 MIPs also appears red. Subsequently, the prepared SiO_2 MIPs was immersed in deionized water. Over time, R6G molecules in SiO_2 MIPs were observed to gradually diffuse into the surrounding deionized water, causing the SiO_2 to fade and the deionized water to appear red. This result confirmed that the template molecules were successfully fixed in SiO_2 MIPs during the preparation process. At the same time, it also shows that R6G and SiO_2 are bonded by weak interaction rather than covalent bond. And there are a large number of pores in SiO_2, which can help R6G molecules diffuse from the inside of the material into the water. Figure S4 illustrates these processes visually.

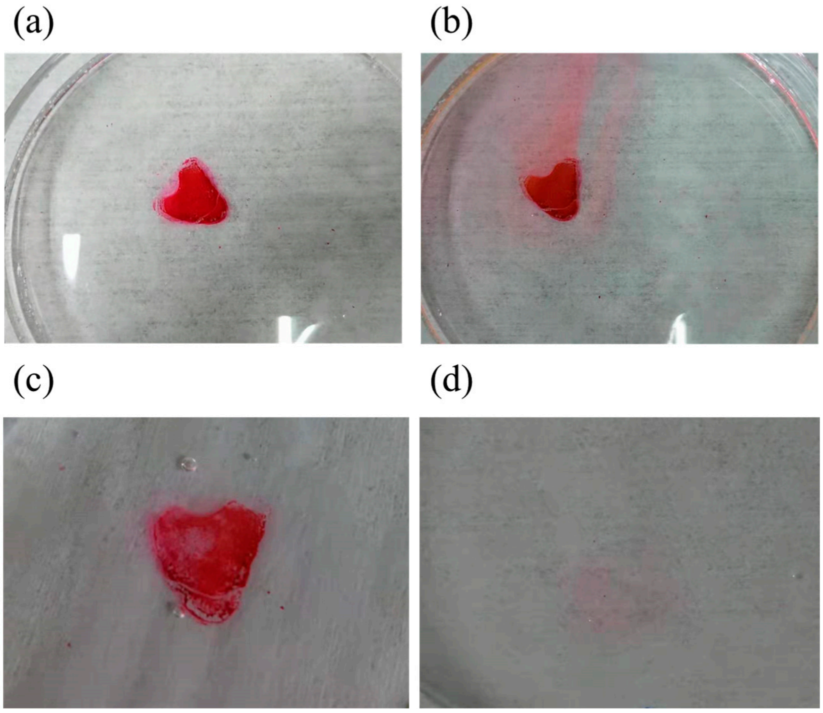

Figure S4. (a) SiO_2 MIPs prepared using R6G as template. (b) The R6G in SiO_2 MIPs diffuses from the material to the water. (c) and (d) The red color of SiO_2 MIPs faded gradually.

For SiO_2 MIPs with DMMP as template, DMMP in materials can be detected by Raman and infrared spectra, whcih are shown in figure S5 and S6, respectively.

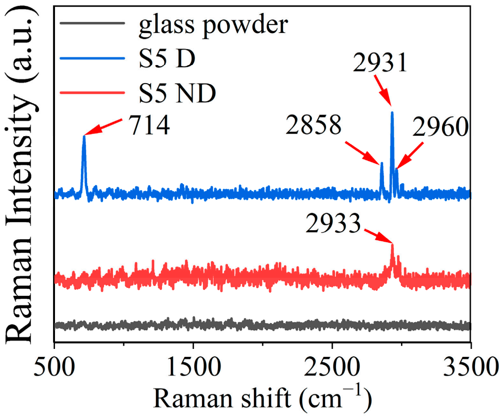

Figure S5. Raman spectra of SiO_2 materials.

As shown in figure S5, using common glass powder as a reference sample, the Raman spectra showed no sign of DMMP peak in glass powder. The Raman spectra of the S5 D sample showed four characteristic DMMP peaks at 714 cm^{-1}, 2858 cm^{-1}, 2931 cm^{-1} and 2960 cm^{-1}, which indicate that DMMP was fixed in SiO_2. For S5 ND sample, the peak intensity of Raman spectrum is very weak due to the elution of DMMP in the material by ethanol. The sample has a very weak peak at 2933 cm^{-1}, which corresponds to the C-H vibration, confirming that a small amount of organic matter remains in the material [36-38].

Figure S6 shows the infrared absorption spectra of samples without elution of DMMP, and significant absorption peaks of DMMP can be observed in S4 D and S5 D samples. Due to the low content of DMMP in S1 D, S2 D and S3 D, the corresponding infrared absorption spectra are easy

to be ignored because of the low intensity.

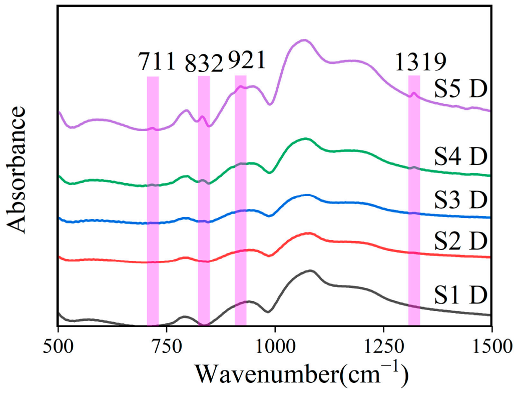

Figure S6. Infrared absorption spectra of SiO_2 MIPs.

After elution of DMMP, the sensitive material prepared by 5 formulations was coated on the SAW device to prepare the corresponding gas sensor. Figure S7 (a) shows the dynamic response curve of 5 gas sensors to DMMP. Figure S7 (b) shows the relationship between sensor response and DMMP concentration. Notably, the S4 sample showed the highest sensitivity over the entire concentration range.

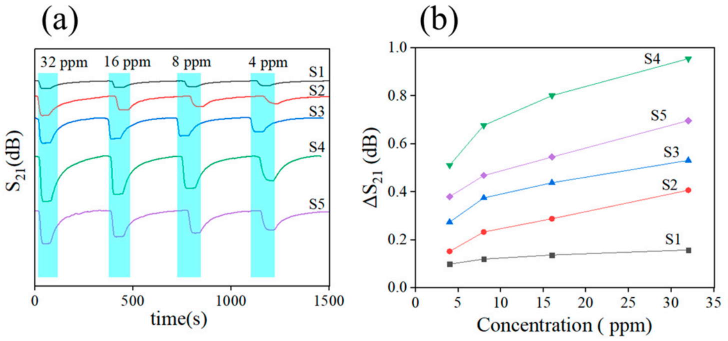

Figure S7. (a)Response curves of the sensors corresponding to the five formulations. (b)The relationship between concentration and response.

The selectivity of the gas sensor is an important parameter, and nine typical gases with a concentration of 300 mg/m^3 were selected to test the responsiveness of sensor. Figure S8 shows the dynamic response curve of the sensor to different gases.

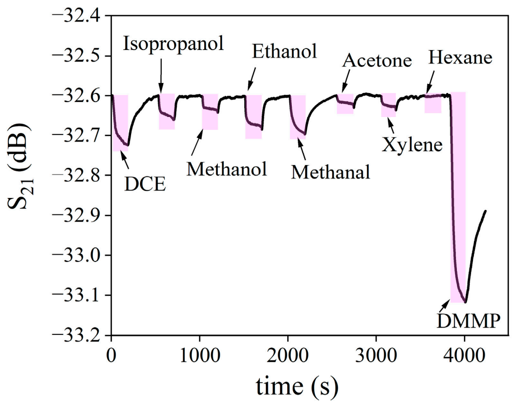

Figure S8. The dynamic response curve of the sensor to different gases.

Table S1. Mass concentration (300 mg/m^3) corresponding volume concentration (ppm)

Compounds	Mass concentration (mg/m^3)	Volume concentration (ppm)
DMMP	300	54
DCE	300	47
Isopropanol	300	112
Methanol	300	209
Ethanol	300	146
Methanal	300	224
Acetone	300	116
Xylene	300	63
Hexane	300	78

The response curves of the sensor under varying humidity are depicted in figure S9. A two-step approach was employed to investigate the humidity on the response of sensor. In the initial stage, a specific volume of water was injected into the evaporator within the chamber to establish a stable humidity level. Subsequently, liquid DMMP was injected into the evaporator to generate DMMP gas. And then evaluate the response of sensor at this humidity level. The test results show that the response of sensor to DMMP decreases with the increase of humidity.

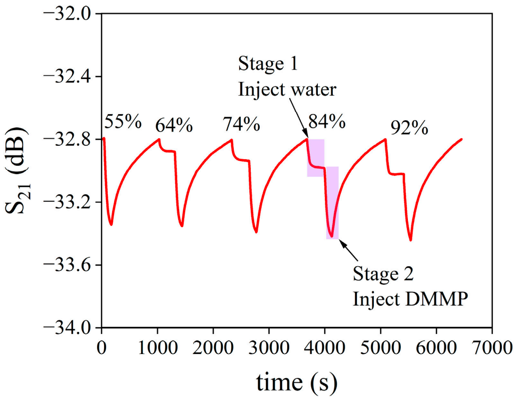

Figure S9. Response curve of the sensor to DMMP at different humidity.

Table S2. Binding energy between silanol and DMMP at different sites

Binding site	DMMP energy (kJ/mol)	Silanol energy (kJ/mol)	Total energy (kJ/mol)	Binding energy (kJ/mol)
D_{O1} - Si_{-H}	-1802843.24	-1556778.05	-3359673.51	-52.23
D_{O2} - Si_{-H}	-1802843.24	-1556778.05	-3359656.24	-34.96
D_{O3} - Si_{-H}	The bimolecular system is unstable			
D_{methyl} - Si_{-O-}	The bimolecular system is unstable			

Table S3. Binding energy of SiO_2 and DMMP

DMMP energy (kJ/mol)	SiO_2 energy (kJ/mol)	Total energy (kJ/mol)	Binding energy (kJ/mol)
-1802502.3	-8277250.43	-10079902.92	-150.19
